# Papillary squamotransitional cell carcinoma of the uterine cervix: a case report and review of the literature

**DOI:** 10.1186/s13256-019-2217-2

**Published:** 2019-10-27

**Authors:** Georgios Gitas, Kubilay Ertan, Achim Rody, Sascha Baum, Dimitrios Tsolakidis, Ibrahim Alkatout

**Affiliations:** 1 Department of Gynecology and Obstetrics, University Hospitals Schleswig Holstein, Campus Luebeck, Ratzeburger Allee 160, House 40, 23538 Luebeck, Germany; 2 Department of Gynecology and Obstetrics, Leverkusen Municipal Hospital, Am Gesundheitspark 11, 51375 Leverkusen, Germany; 3 1st Department of Obstetrics and Gynecology, Papageorgiou General Hospital, Aristotle University of Thessaloniki, Thessaloniki, Greece; 4 Department of Gynecology and Obstetrics, University Hospitals Schleswig Holstein, Campus Kiel, Arnold-Heller-Strasse 3, House 24, 24105 Kiel, Germany

**Keywords:** Rare cervical cancer, Transitional cell carcinoma, Uterine cervix

## Abstract

**Background:**

Papillary squamotransitional cell carcinoma of the uterine cervix is a rare neoplasm, a subtype of transitional cervical carcinoma that appears to be a variation of squamous cervical carcinoma. It has a disposition toward metastasis at an advanced stage and local recurrence. Owing to the difficulty of illustrating the invasion histologically, misdiagnosis is likely to affect the patient’s prognosis.

**Case presentation:**

We present a case report of an 81-year-old Caucasian patient with squamotransitional cell carcinoma with unusual clinical behavior that was primarily thought to be ovarian cancer. According to the clinical examination and radiologic imaging, the patient had no vaginal bleeding and a normal cervix. Nevertheless, the tumor was already metastasized at the retroperitoneal tissue and at the right ovary. Computed tomography-guided biopsy of the right adnexa gave no further clarification. Although the tumor resembled urothelial cancer, this diagnosis was dismissed because of the results of immunohistochemistry analysis with CK7^+^, CK5^+^, and CK20^−^. Because of the differential diagnosis of ovarian cancer, we decided in favor of an exploratory surgical approach. Hysterectomy with bilateral adnexectomy, extensive retroperitoneal tumor debulking, and infragastric omentectomy was performed by laparotomy. Histopathology revealed a squamotransitional cervical cancer as the primary tumor with a tumor stage of pT3b, pN1 (1/2), V0, RX, G2, corresponding to International Federation of Gynecology and Obstetrics stage IIIB.

**Conclusions:**

As far as we are aware, this is the first report of papillary squamotransitional cell carcinoma of the uterine cervix metastatic to the ovary without vaginal bleeding and with a clinically and radiologically unsuspicious cervix. Physicians should always contemplate papillary squamotransitional cell carcinoma of the uterine cervix in unclear cases with ovarian metastasis, especially if the histology indicates a transitional cancer (CK7^+^ and CK20^−^), before proceeding with treatment. More cases are needed to illuminate the clinical characteristics and categorization of papillary squamotransitional cell carcinoma of the uterine cervix.

## Background

Papillary cell carcinomas of the uterine cervix are very rare but histologically clearly described with well-known histopathological features [1, 2]. To the best of our knowledge, there is no exact information about the incidence of papillary squamotransitional cell carcinoma (PSTCC). This type of cancer may show either a transitional, squamous, or mixed differentiation. PSTCC is named as such because it presents a mixed spectrum of morphologies with a more transitional or more squamous histological appearance [1–3]. PSTCC can be described as an *in situ* tumor with or without an invasive part but usually with both components existing. Furthermore, it is difficult to distinguish PSTCC from other papillary lesions of the cervix because of its papillary, exophytic nature. There is a disposition toward late metastasis at an advanced stage and local recurrence [2].

Transitional cell carcinomas were first described in the ovaries and fallopian tubes and many years later were observed in the uterine cervix and endometrium [4, 5]. These tumors were initially identified in 1952 by Marsh and characterized as papillary squamous cell carcinomas in 1986 by Randall *et al.* [6, 7]. Information on the clinical, histological, and immunohistochemical features of this tumor is based on a few case reports and a small case series in the literature [8]. Because of the rarity of this tumor and consequently the limited data, no information is available on tumors with clinical behavior similar to that of our patient’s case.

We present an unprecedented case of PTSCC with unusual clinical appearance. All cases of PSTCC should be reported to improve understanding of this rare tumor. Further observation of this and other cases is important to achieve a better comparison of the biological and clinical behavior of this tumor. Despite the rare incidence of PTSCC, the gynecologist should be aware of this tumor as an important differential diagnosis in women with genital cancer, especially when the histology indicates a transitional cancer, in order to provide the appropriate treatment.

## Case presentation

**Fig. 1:**
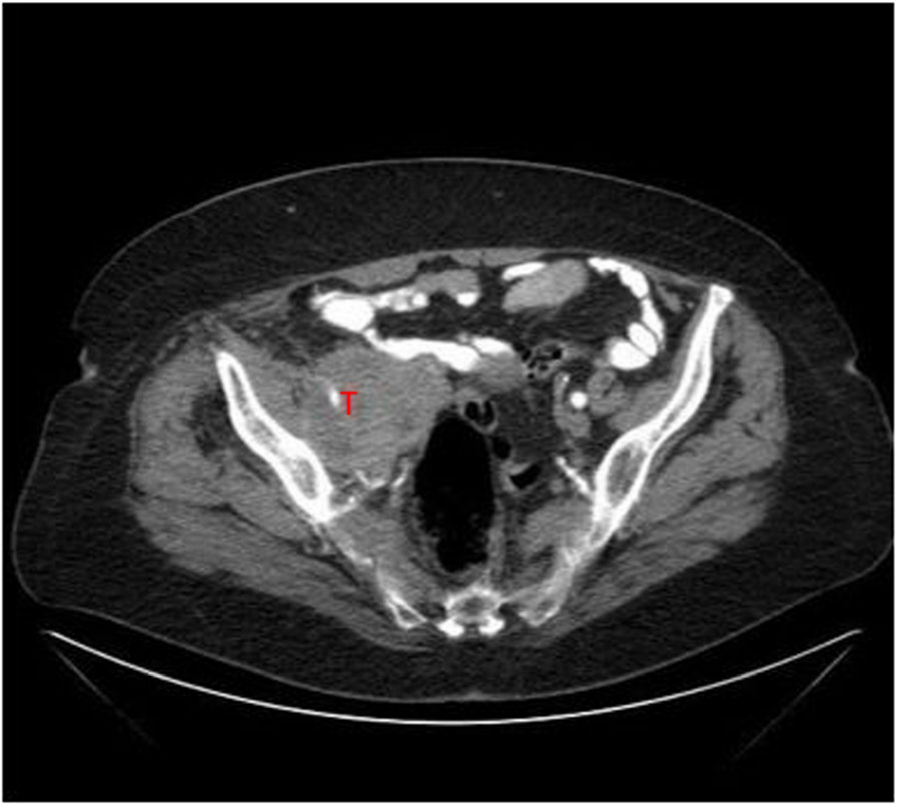
Transverse abdominal computed tomographic scan. *T* Tumor

**Fig. 2:**
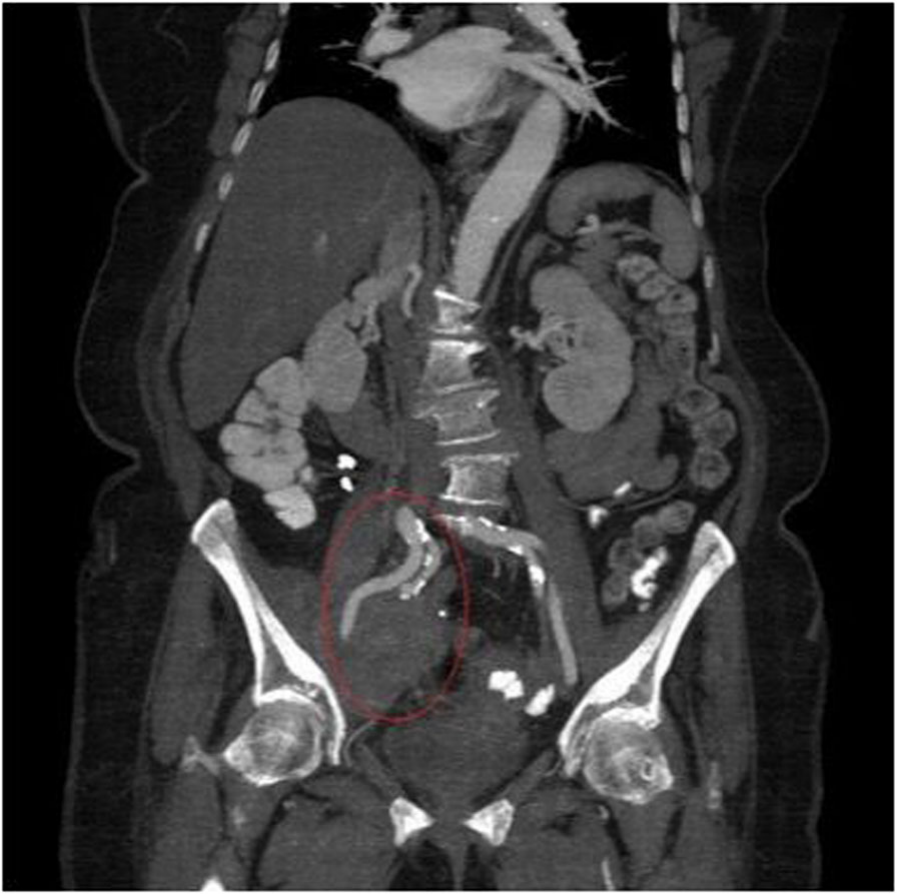
Frontal or coronal abdominal computed tomographic scan. The tumor lies on the external iliac artery (encircled)

We present a case of an 81-year-old Caucasian patient with a retroperitoneal tumor on the right side of the pelvis diagnosed by computed tomography (CT) during her stay in the hospital for deep vein thrombosis of the right leg. Abdominal CT revealed a tumor, which was first thought to be ovarian cancer, sized 7.5 cm × 6 cm × 6 cm with light inhomogeneous density at the right ovary (Fig. 1), lying on the external iliac artery (Fig. 2) and infiltrating the neighboring muscles at the pelvic wall. No distant metastasis or ascites was observed. The patient was then transferred to our gynecological division with the suspicion of primary ovarian cancer.

The general health condition of the patient was good for her age. Her past medical history revealed a spontaneous vaginal delivery and no operations. In her family history, there was no evidence of cervical or any other type of cancer. Furthermore, the patient did not smoke or consume alcohol. She had hypertension, which was being treated with a β-blocker; Hashimoto’s thyroiditis, which was being treated with levothyroxine; and pernicious anemia, which was being treated with vitamin B_12_ substitution. The patient was raised in Germany, which has an established high standard of living and a well-developed social security system.

The patient did not present with any unusual pain or vaginal bleeding. Her clinical examination and laboratory findings, including complete blood count, urine analysis, and liver and renal function, were normal, except for a palpable mass on the right side of the pelvis. On admission, the patient’s vital parameters were normal (temperature 36.5 °C, pulse 60 per minute, blood pressure 85/140 mmHg). Furthermore, the results of her gynecological clinical examination, including transvaginal ultrasound and visual inspection of the cervix and the vagina, were completely normal. Upon palpation, the uterus was mobile, and the tumor at the right adnexa was almost fixed.

The patient’s ultrasound examination revealed no suspicious results for endometrial or cervical cancer, but at the right adnexa, an irregular restricted tumor, 10 × 6 × 8 cm in size, was found. The tumor marker CA 125 was pathological with 133 U/ml (normal value < 35 U/ml).

Because the spread of the tumor was unusual for ovarian cancer, we decided to proceed with a CT-guided biopsy of the right adnexa. This revealed a solid cancer infiltrating the right ovary and resembling a cancer of the urothelium. Immunohistochemistry (IHC) analysis revealed expression of cytokeratin 7 (CK7^+^) and cytokeratin 5 (CK5^+^) but a negative finding for cytokeratin 20 (CK20^−^). The estrogen receptors of the tumor were completely negative, and the reaction for p63 was recorded as positive. A neoplastic ureteral disease was excluded after the use of diagnostic cystoscopy and pyelography. Because of the differential diagnosis of ovarian cancer, we finally decided in favor of an exploratory surgical approach.

As expected, intraoperatively, a tumor (10 cm) was found in the right retroperitoneal space, emerging from the edge of the ovary and reaching the uterus. The remainder of the peritoneum, bowel, pelvic and paraaortic lymph nodes, and upper abdominal organs were macroscopically unsuspicious. Hysterectomy with bilateral adnexectomy, extensive retroperitoneal tumor debulking, and infragastric omentectomy were performed by laparotomy. The right external iliac vein was completely obstructed because of the tumor’s infiltration, and the right external iliac artery, which could be completely preserved, had stenosis. The entire venous blood flow of the right leg ran through the internal iliac vein. The results of a frozen section revealed transitional cancer with infiltration of part of the right ovary. During the operation, the primary origin of the tumor could not be recognized. The size of residual disease was less than 5 mm. A radical operation was not indicated, owing to the advanced age of the patient and its association with serious complications, such as compromised venous circulation of the right leg with the increased risk of mortality.

**Fig. 3:**
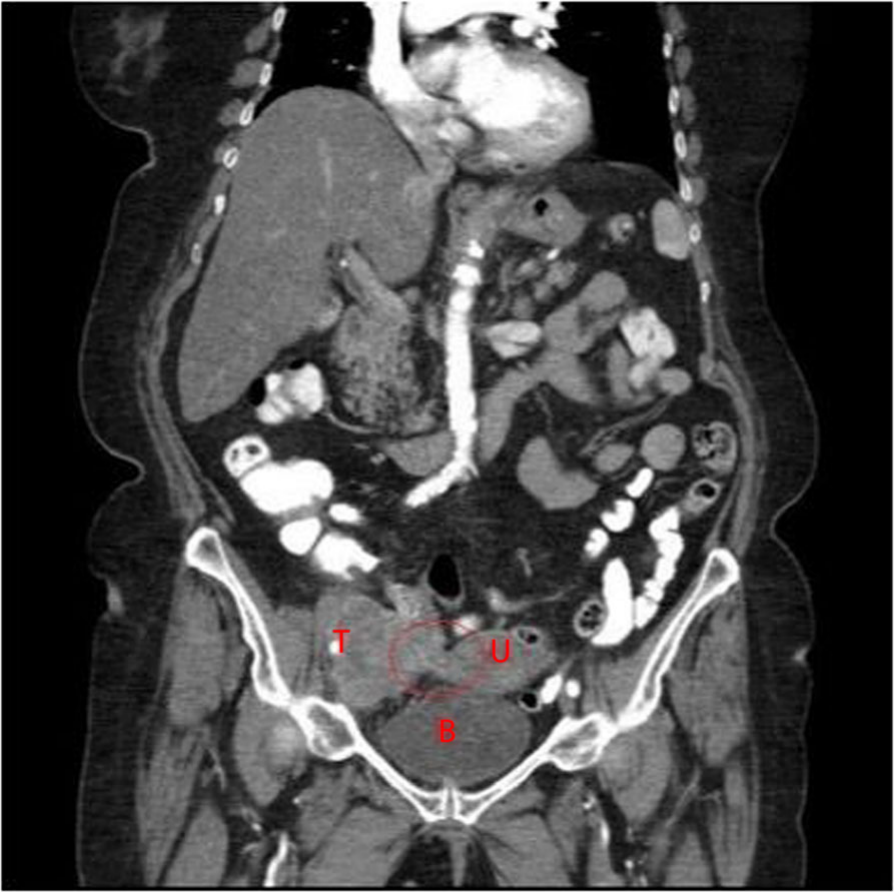
Coronal abdominal computed tomographic scan. *B* Bladder, *T* Tumor, *U* Uterus. Retrospectively, a tissue connection between retroperitoneal tumor and cervix could be recognized

The histopathologic analysis results revealed, as the primary tumor, a squamotransitional cervical cancer with a histopathologic tumor stage of pT3b, pN1 (1/2), V0, R1, G2, corresponding to International Federation of Gynecology and Obstetrics (FIGO) stage IIIB. Retrospectively, we could observe a connection between retroperitoneal tumor and cervix on a CT scan (Fig. 3).

**Fig. 4:**
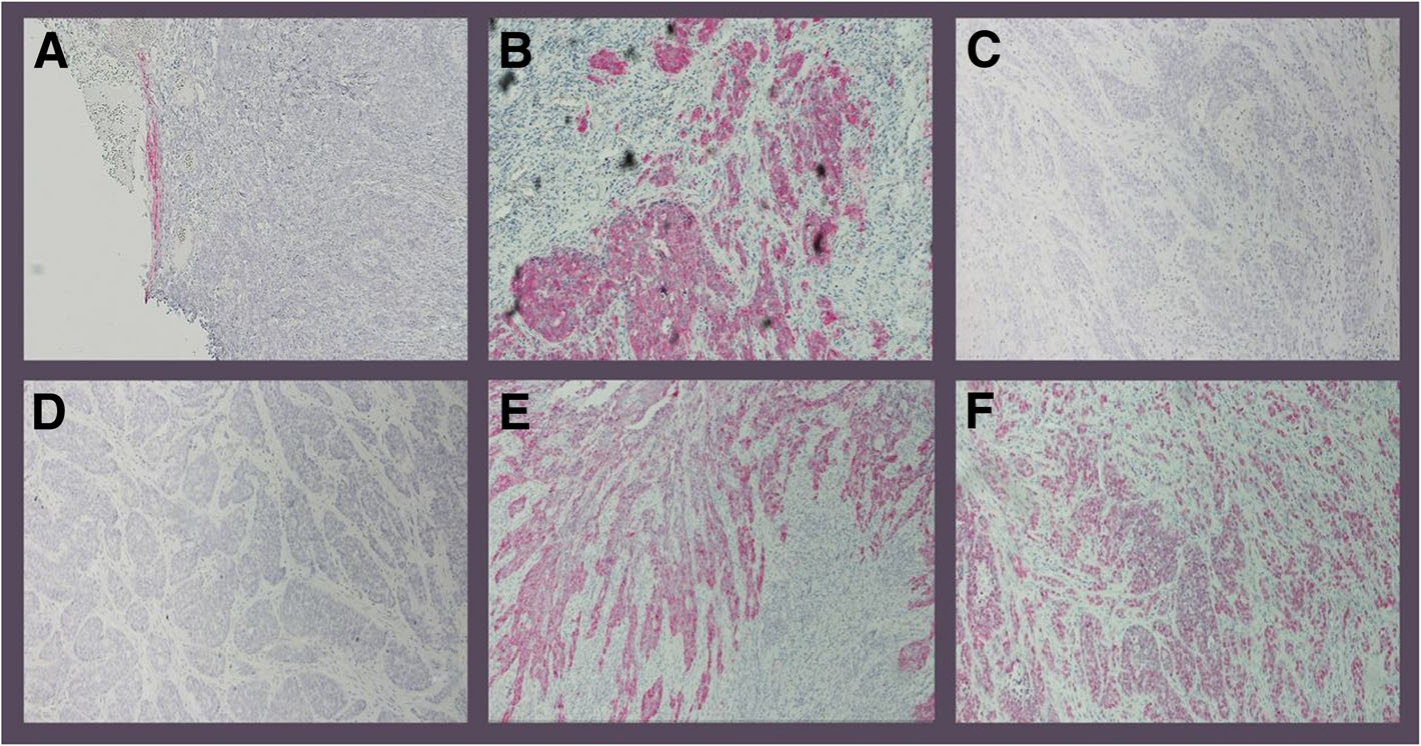
Immunohistochemistry (IHC) analysis with positive staining reaction for CK14 (**a**), positive staining reaction for p16 (**b**), negative staining reaction for chromogranin (**c**), negative staining reaction for synaptophysin (**d**), expression of cytokeratin 7 (**e**), and positive staining reaction for p63 (**f**)

Macroscopic examination revealed that the cervix had a gray-white color and a smooth surface. Retention cysts were also located in the cervix. Microscopic examination showed that the surface of ​​the ectocervix was covered with nonkeratinizing, stratified squamous epithelium. In the area of the transformation zone, atypical epithelial formations were found with moderate cellular and nuclear polymorphism. The tissue of the tumor of the right retroperitoneum had epithelial atypia with uncontrolled cell growth and infiltration of the trabecular formations. IHC revealed positive staining for CK14, p16, and p63 and negative staining for chromogranin and synaptophysin (Fig. 4).

We concluded that radiotherapy with 60 Gy at the former tumor region and 54 Gy at the pelvic lymphatic system was required; however, the patient refused further treatment, including the follow-up examinations.

Five months later, the patient was hospitalized with an unclear infection. A CT scan of the abdomen revealed a huge abscess in the right retroperitoneal space and recurrence of the tumor. After treatment with pigtail tube drainage and antibiotics, we were able to stabilize her condition. Subsequently, the patient refused any further medical treatment, so she was transported to the palliative unit, where she died a few days later of sepsis.

## Discussion and conclusions

We describe an unusual case of a patient with PTSCC who underwent surgery because of the suspicion of primary ovarian cancer based on a 7.5-cm tumor at the right ovary. The patient did not present with any unusual pain or vaginal bleeding. Moreover, the result of the patient’s clinical examination, including transvaginal ultrasound and visual inspection of the cervix and the vagina, was completely normal. CT-guided biopsy, intraoperative frozen section, and exploratory laparotomy were unable to clarify the origin of the tumor. The final histopathologic result revealed PTSCC with tumor stage pT3b, pN1 (1/2), V0, RX, G2, corresponding to FIGO stage IIIB.

It remains unclear whether papillary cell carcinomas of the cervix represent a different group of tumors or if they are morphologically similar within a single clinically homogeneous entity. Koenig *et al.* [2], who presented 32 cases of papillary cervical carcinomas, the largest case series in the literature, reported that the most common clinical presentation of PSTCC is abnormal bleeding and an abnormal Papanicolaou smear [2]. With regard to our patient’s case, a cervical biopsy was not performed, because the result of visual inspection of both the cervix and the vagina was completely normal. In addition, because the retroperitoneal tumor was at an advanced stage, cervical cancer as the primary tumor was not expected. Even in the case of clinical, sonographic, or radiologic suspicion of cervical cancer, it is not always easy to identify this neoplasm, owing to its papillary, exophytic nature, and sometimes a deep biopsy or a cone biopsy is necessary [1–3, 7].

PSTCC may exist in an *in situ* state with or without an invasive part, but usually it exhibits both components [2]. The invasive component is mostly without papillae, in contrast to the noninvasive component, which is more often papillary. It has been reported that PSTCC can be understaged because it may be difficult to illustrate histologically the invasion part, which can lead to incorrect staging, affecting the patient’s treatment and prognosis [3, 15]. As a result, it is very important to distinguish PSTCC from other papillary lesions of the cervix (Table 1). Furthermore, the papillary growth pattern makes it similar to a carcinoma of urothelial origin [10, 15, 16]. This diagnosis was suspected in our patient’s case, and our patient underwent cystoscopy to exclude urothelial carcinoma. As shown in Table 2, transitional cell carcinoma of urothelial origin often reveals a different immunoreactivity [12–14], which in our patient’s case helped us to dismiss this diagnosis. The uterine cervix must always be considered as the site of origin of transitional carcinoma.

**Table 1:** Differential diagnosis of papillary lesions of the cervix

Benign	Malignant
Condyloma acuminatum	Warty squamous cell carcinoma
Squamous papilloma	Verrucous carcinoma
Cervical intraepithelial neoplasia with papillary configuration	Transitional cell carcinoma of the endometrium and endometrial adenocarcinoma
Papillary adenofibroma	Villoglandular papillary adenocarcinoma

Papillary squamotransitional cell carcinomas of the uterine cervix (PSTCC) have a papillary protrusion bulging with fibrovascular cores under a few layers of atypical epithelial cells, resembling a high-grade squamous intraepithelial lesion of the cervix. Condylomata and papillomas present an endophytic growth pattern with trabeculae of transitional cells and anastomosing cords. To differentiate these lesions, mitotic activity, cellularity, and cytologic atypia expression, which increase in the case of PSTCC, are examined [14, 17]

**Table 2:** Immunohistochemistry (IHC) staging with antigens (markers) for the differential diagnosis between cervical and urothelial cancer

Marker	PSTCC	SCC	TCC of urothelial origin
CK7	+	+	+
CK20	–	–	+
CK19	++/−	++/−	+
P53	+	+	+
Uroplakin III	–	–	+
Ki67	+	+	+

*PSTCC* Papillary squamotransitional cell carcinoma of the uterine cervix, *SCC* Squamous cell carcinoma of the uterine cervix, *TCC* Transitional cell carcinoma of the bladder

Some reports advocate the hypothesis that the majority of transitional cell carcinomas of the cervix are variations of squamous cervix carcinoma (SCC) because they share almost the same etiologic and pathogenic features, such as human papillomavirus (HPV) infection, an immunoreactive profile, spread, and behavior, with the exception that late metastasis has been reported in referring to PSTCC [11]. Many reports describe that PSTCC has a positive correlation with HPV16 and a negative correlation with HPV6, 11, and 18. This finding supports the above-mentioned hypothesis that PSTCC and SCC are similar and suggests that HPV infection of the cervix may play an etiologic role in at least some of these tumors [2, 9, 14]. Currently, there are no specific treatment guidelines for PSTCC [18]; however, experts suggest the same treatment as for SCC [2, 7]. Koenig *et al.* [2] reported longer survival in one patient with advanced disease after treatment with chemotherapy. More cases are needed to clarify the appropriate treatment of this rare tumor.

In the literature, the clinical behavior of PSTCC is characterized as almost the same as that of SCC, except for the tendency of PSTCC toward late metastasis and local recurrence [2, 7]. However, in our patient’s case, the clinical behavior seems to have been fundamentally different, because the tumor extended into the retroperitoneal space and the right ovary without apparent local infiltration of the cervix or vaginal bleeding. The treatment of the patient would have been the same if we had known from the beginning that she had PSTCC. In the case of young patients, however, the treatment could affect the recurrence of the tumor and the postoperative survival of the patient if the correct diagnosis is not made during staging and as a result the appropriate oncologic procedure and adjusted therapy are not initiated.

In conclusion, to the best of our knowledge, this is the first report of a retroperitoneal PSTCC metastatic to the ovary without vaginal bleeding and with a normal cervix at clinical examination and imaging. Such a case can easily be misdiagnosed as primary ovarian cancer because of the absence of any visible cervical lesion or abnormal cervical screening history. A high index of suspicion is necessary to avoid misdiagnosis of such a case and to choose the appropriate treatment, which could strongly influence the patient’s prognosis. Thus, physicians should be aware of this case and should always consider PSTCC in patients with ovarian metastasis of unclear diagnosis despite an unsuspicious cervix, especially when the histology indicates a transitional cancer (CK7^+^ and CK20^−^). Further observation of this and other cases is important to achieve a better comparison of the biological and clinical behavior of this tumor.

## Data Availability

The datasets used and analyzed during the current study are available from the corresponding author on reasonable request.
